# Implementing health research through academic and clinical partnerships: a realistic evaluation of the Collaborations for Leadership in Applied Health Research and Care (CLAHRC)

**DOI:** 10.1186/1748-5908-6-74

**Published:** 2011-07-19

**Authors:** Jo Rycroft-Malone, Joyce E Wilkinson, Christopher R Burton, Gavin Andrews, Steven Ariss, Richard Baker, Sue Dopson, Ian Graham, Gill Harvey, Graham Martin, Brendan G McCormack, Sophie Staniszewska, Carl Thompson

**Affiliations:** 1 Centre for Health-Related Research, School of Healthcare Sciences, Bangor University, Bangor, Gwynedd, UK; 2 Faculty of Social Sciences, McMaster University, Hamilton, Ontario, Canada; 3 ICOSS, School of Health & Related Research, University of Sheffield, Sheffield, UK; 4 Department of Health Sciences, University of Leicester, Leicester, UK; 5 Said Business School, University of Oxford, Oxford, UK; 6 Canadian Institutes of Health Research, Elgin Street, Ottawa, Ontario, Canada; 7 Manchester Business School, University of Manchester, Manchester, UK; 8 Department of Health Sciences, University of Leicester, Leicester, UK; 9 Institute of Nursing Research, University of Ulster, Coleraine, Co. Londonderry, N. Ireland; 10 School of Health & Social Studies, University of Warwick, Coventry, UK; 11 Department of Health Sciences, University of York, Heslington, York, UK

## Abstract

**Background:**

The English National Health Service has made a major investment in nine partnerships between higher education institutions and local health services called Collaborations for Leadership in Applied Health Research and Care (CLAHRC). They have been funded to increase capacity and capability to produce and implement research through sustained interactions between academics and health services. CLAHRCs provide a natural 'test bed' for exploring questions about research implementation within a partnership model of delivery. This protocol describes an externally funded evaluation that focuses on implementation mechanisms and processes within three CLAHRCs. It seeks to uncover what works, for whom, how, and in what circumstances.

**Design and methods:**

This study is a longitudinal three-phase, multi-method realistic evaluation, which deliberately aims to explore the boundaries around knowledge use in context. The evaluation funder wishes to see it conducted for the process of learning, not for judging performance. The study is underpinned by a conceptual framework that combines the Promoting Action on Research Implementation in Health Services and Knowledge to Action frameworks to reflect the complexities of implementation. Three participating CLARHCS will provide in-depth comparative case studies of research implementation using multiple data collection methods including interviews, observation, documents, and publicly available data to test and refine hypotheses over four rounds of data collection. We will test the wider applicability of emerging findings with a wider community using an interpretative forum.

**Discussion:**

The idea that collaboration between academics and services might lead to more applicable health research that is actually used in practice is theoretically and intuitively appealing; however the evidence for it is limited. Our evaluation is designed to capture the processes and impacts of collaborative approaches for implementing research, and therefore should contribute to the evidence base about an increasingly popular (*e.g.*, Mode two, integrated knowledge transfer, interactive research), but poorly understood approach to knowledge translation. Additionally we hope to develop approaches for evaluating implementation processes and impacts particularly with respect to integrated stakeholder involvement.

## Background

Despite considerable investment in the generation of research, for the most part it is not routinely used in practice or policy [1-4]. In the United Kingdom (UK), a national expert group reviewed the implementation research agenda and recommended sustained and strategic investment in research and infrastructure aimed at increasing our capability and capacity to maximise the impact of health research [5]. The group also recommended that implementation researchers and implementation research should be embedded within health services [6-11]. In response to the recommendations of Clinical Effectiveness Research Agenda Group (CERAG), there has been a major investment in nine partnerships between higher education institutions and local health services within the English National Health Service (NHS) [12,13]. The Collaborations for Leadership in Applied Health Research and Care (CLAHRC) are funded by the National Institute for Health Research (NIHR) to produce and implement research evidence through sustained interactions between academics and services (see Additional File 1 for more information about the CLAHRC concept). The establishment of the CLAHRCs and their explicit remit for closing the gap between research and practice provides a natural 'experiment' for exploring and evaluating questions about research implementation within a partnership model. This protocol describes one of four externally funded evaluations of CLAHRC (NIHR SDO 09/1809/1072).

### Implementing research in practice

Health services are more or less informed by the findings of research [14-19]. The Cooksey Report [20] distinguishes between two gaps in knowledge translation: the 'first' gap between a scientist's bench to product/process/service, and the 'second' gap, their routine use in practice. It is the second gap that has been neglected and provides the focus for our evaluation. Specifically, we are interested in exploring implementation in its broadest sense. This breadth includes acknowledging that information and knowledge comes in many forms, such as research, audit data, patient and public involvement, and practice know how, which variably inform decision making and service delivery. We treat research implementation and knowledge translation as related concepts, sharing a largely common literature and theory base. Both concern closing the gap between what is known from research and implementation of this by stakeholders pursuing improved health outcome and experiences.

Implementation is a slow, complex and unpredictable process [14,15,21-27]. The rational-logical notion that producing research, packaging it in the form of guidelines and assuming it will automatically be used is now outdated. There is a substantial body of evidence showing that using research involves significant and planned change involving individuals, teams, organisations and systems [14,22-24,28-33]. One meta-synthesis of case studies showed that adopting knowledge depends on a set of social processes that include sensing and interpreting new evidence, integrating it with existing evidence; reinforcement (or not) by professional networks, which in turn is mediated by local context [23], including the contribution that patients and the public make.

Context is emerging as a significant influence on knowledge flow and implementation. Micro, meso and macro contextual influences [34] include factors such as financial and human resources [14,15,31], structure [22], governance arrangements [31], culture [27,35-38], power [38,39], and leadership [22,23,28,33,35,40]. Such factors appear to influence an organisation's capacity to manage, absorb, and sustain knowledge use [26]. However we do not know whether some contextual factors are more influential than others, or how they operate and change over time.

Networks and communities of practice [41] may also play an important role in both the flow and use of evidence [14,23,41-45]. Multi-disciplinary communities of practice have been found to transform research evidence through interaction and collective sense making, such that other forms of knowledge (*e.g.*, practice know how) become privileged [44,45]. Whilst communities of practice are intuitively appealing, there is little empirical research to support claims that they actually increase knowledge uptake in health services [46-48]. There is evidence to suggest that communities of practice show promise as a means of creating and sharing knowledge that has meaning for practitioners [49], however little is known about the mechanisms by which this may occur. There is an opportunity within this study to explore the relevance of communities of practice to the implementation of research, what mechanisms and processes may be at work, and the role that patients and the public may play in this.

'Boundary objects' may facilitate or inhibit knowledge flow [50-54]. Typically boundary objects are representations, abstractions, or metaphors that have the power to 'speak to' different communities of practice by sharing meaning and learning about each others' perspectives and by acting as (temporary) anchors or bridges [50-54]. The theory of 'boundary objects' has importance in exploring the translation of meaning from one setting to another. Objects have the capability to be understood by actors in more than one setting, for example, between different departments, doctors and nurses, researchers and users, and practitioners and patients. We are interested in finding out whether such boundary objects exist in the CLAHRCs and the NHS communities they serve-and if they do, what do they look like and how are they being used, particularly in relation to implementation.

## Summary

To date, funders and policy makers have focused on the generation of research knowledge to the relative neglect of how research is used in practice. A number of NHS initiatives including Academic Health Science Centres, Health Innovation and Education Clusters, and Quality Observatories are emerging that could help bridge research and practice. However the CLAHRCs have an explicit remit for closing the gap in translation. Implementation has generally been studied through one-off, retrospective evaluations that have not been adequately theorised, which leaves many questions unanswered. This study is a theory driven, longitudinal evaluation of research implementation within CLAHRCs and will address some critical gaps in the literature about increasing applied health research use.

### Study objectives

We are exploring how research is implemented within CLAHRCs through the following aims and objectives.

### Aims

The aims of this study are:

1. To inform the NIHR SDO programme about the impact of CLAHRCs in relation to one of their key functions: 'implementing the findings from research in clinical practice.'

2. To make a significant contribution to the national and international evidence base concerning research use and impact, and mechanisms for successful partnerships between universities and healthcare providers for facilitating research use.

3. To work in partnership so that the evaluation includes stakeholder perspectives and formative input into participating CLAHRCs.

4. To further develop theory driven approaches to implementation research and evaluation.

### Objectives

The objectives of this study are:

1. To identify and track the implementation mechanisms and processes used by CLAHRCs and evaluate intended and unintended consequences (*i.e.*, impact) over time.

2. To determine what influences whether and how research is used or not through CLAHRCs, paying particular attention to contextual factors.

3. To investigate the role played by boundary objects in the success or failure of research implementation through CLAHRCs.

4. To determine whether and how CLAHRCs develop and sustain interactions and communities of practice.

5. To identify indicators that could be used for further evaluations of the sustainability of CLAHRC-like approaches.

### Theoretical framework

**Figure 1:**
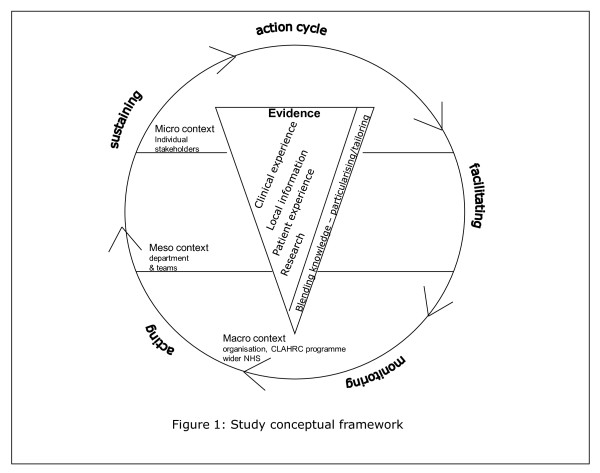
**Conceptual framework**.

Implementation research has tended to lack a theoretical basis [32,55,56] and has been described as an 'expensive version of trial and error' [32]. For this study, our overarching conceptual framework reflects the complexities of research implementation (Figure 1) and draws on the Promoting Action on Research Implementation in Health Services (PARIHS) [15,37,57,58] and Knowledge to Action (KTA) [17] frameworks. PARIHS represents the interplay of factors that play a role in successful implementation (SI); represented as a function (f) of the nature and type of evidence (E), the qualities of the context (C) in which the evidence is being used, and the process of facilitation (F); SI = f(E,C,F). The KTA framework is underpinned by action theory and stakeholder involvement, containing a cycle of problem identification, local adaptation and assessment of barriers, implementation, monitoring, and sustained use. The frameworks complement each other: PARIHS provides a conceptual map, and the KTA framework is an action-orientated understanding of knowledge translation processes. Our conceptual framework provides a focus for what we will study (*e.g.*, qualities and perceptions of evidence, contextual influences, approaches and dynamics of implementation) and for integrating data across sets and sites. The strength of our conceptual framework is that it is based on knowledge translation theory but is also flexible enough to be populated by multiple theories at multiple levels.

## Methodology and methods

### Approach

This study is a longitudinal three-phase, multi-method evaluation, which deliberately aims to explore the boundaries between knowledge use in practice. The evaluation, as expressed by the funder, is being conducted for the process of learning, not for judgement. Given the processual and contextual nature of knowledge use and our objectives, realistic evaluation is our overarching methodology [59]. Realistic evaluation is an approach that is underpinned by a philosophy of realism that recognises reality as a construction of social processes. Thus realists attempt to understand complex social interactions/interventions. Complex social interventions according to Pawson and Tilley [60,61] are comprised of theories, involve the actions of people, consist of a chain of steps or processes that interact and are rarely linear, are embedded in social systems, prone to modification and exist in open, dynamic systems that change through learning. As such, realistic evaluation offers a means of understanding network-based approaches such as CLAHRCs, which by their nature are social systems, involve the actions of people and groups, and which are likely to change over time. Realistic evaluation is also a useful approach for capturing contextual influences and changes at multiple levels over time because of the cyclical approach to evaluation.

Others have successfully used realistic evaluation to evaluate complex, system, and network orientated initiatives [*e.g.*, [62,63]] and in implementation related research [64-66]. For example Greenhalgh and colleagues [63] evaluated a whole-system transformation in four large healthcare organisations in London. They identified implementation mechanisms and sub-mechanisms, with associated enabling and constraining factors, which included networks (hard and soft), evidence, structures, contracts, governance, and roles http://axisto.com/webcasting/bmj/berlin-2009/plenary-3/index.htm). Additionally, Sullivan and colleagues [62] successfully used realistic evaluation to evaluate a national initiative in which they specified the types and levels of collaborative activity necessary to deliver Health Action Zone objectives. Rycroft-Malone *et al. *[64-66] conducted a realistic evaluation of the mechanisms and impact of protocol-based care within the NHS. There are growing numbers of researchers engaged in realistic evaluation research (for example [67-69]), this evaluation provides a further opportunity to test and develop the approach.

Within realism, theories are framed as propositions about how mechanisms act in contexts, to produce outcomes. Realistic evaluation is particularly relevant for this study because it aims to develop explanatory theory by acknowledging the importance of context to the understanding of why interventions and strategies work. Programmes (*i.e.*, CLAHRC implementation) are broken down so that we can identify what it is about them (mechanisms) that might produce a change (impact), and which contextual conditions (context) are necessary to sustain changes. Thus, realistic evaluation activity attempts to outline the relationship between mechanisms, context, and outcomes.

We are interested in exploring the various ways that evidence can impact. Therefore within this evaluation we will be focussing on a broad range of outcomes, including:

1. Instrumental use: the direct impact of knowledge on practice and policy in which specific research might directly influence a particular decision or problem.

2. Conceptual use: how knowledge may impact on thinking, understanding, and attitudes.

3. Symbolic use: how knowledge may be used as a political tool to legitimatise particular practices.

4. Process use: changes that result to policy, practice, ways of thinking or behaviour resulting from the process of learning that occurs from being involved in research. [26,70-72].

This proposal has been developed by a team including participants from four CLAHRCs (RB, CT, GH, GM, and SA). Their involvement from the outset ensures the evaluation is addressing questions of interest, is feasible, and offers opportunities for mutual learning and benefit. We recognise that those being evaluated being part of the evaluation team, whilst consistent with an interactive approach [73-77] calls for particular attention to issues of rigour. Sociological and anthropological research, utilisation-focused evaluation, and participant action research have a longstanding tradition of including 'insiders' [78,79]. An insider perspective will provide insight and enable us to crosscheck face validity of data against the experience of operating within a CLAHRC context. Our approach is consistent with the principles upon which the CLAHRCs were created, and the proposed methods have their own criteria for rigour and integrity [80,81]. However, we acknowledge that the evaluation, through its activities and formative input might influence how participating CLAHRCs approach implementation over time. We have therefore built in a process for monitoring any cross fertilisation of ideas and their potential impact (see section below for more information).

### Phases and methods

In keeping with utilisation-focused evaluation principles [82] our plan integrates ongoing opportunities for interaction between the evaluation team, three participating CLAHRCs, and the wider CLAHRC community to ensure findings have programme relevance and applicability.

### Realistic evaluation case studies

The three participating CLARHCS provide an opportunity to study in-depth comparative case studies of research implementation [81]. We have focussed on three CLAHRCs because it would not be practically possible to capture the in-depth data required to meet study aims and objectives across all nine CLAHRCs. However, there are opportunities throughout the evaluation for the wider CLAHRC community to engage in development and knowledge sharing activities (participating CLAHRCs are described in more detail in Additional Files 2, 3 and 4).

A 'case' is implementation [theme/team] within a CLAHRC and the embedded unit, particular activities/projects/initiatives related to a tracer issue [81]. These cases represent a natural sample of the CLAHRCs as each has planned a different approach to implementation. Sampling is based on a theoretical replication argument; it is anticipated that each CLAHRC will provide contrasting results, for predictable reasons [81].

**Figure 2:**
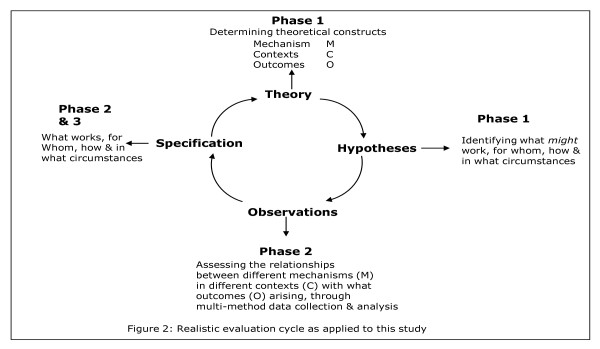
**Realistic Evaluation Cycle**.

To facilitate studying research implementation in depth, within each case we will focus on three knowledge pathways (embedded unit of analysis), which will become 'tracer' issues (further description below). With each tracer issue, there will be a community of practice, a group of people with a shared agenda, who pool expertise, and gather and interpret information to meet objectives which may include knowledge producers, implementers, and users. The realistic evaluation cycle represents the research process as hypotheses generation, hypotheses testing and refining (over several rounds of data collection), and programme specification as shown in Figure 2. These phases are described below.

### Phase one: Hypotheses generation (up to 18 months)

In this first phase, we will: develop good working relationships and establish ways of working with participating CLAHRCs; develop an evaluation framework that will provide a robust theoretical platform for the study; and map mechanism-context-outcome (MCO) links and generate hypotheses, *i.e.*, what might work, for whom, how, and in what circumstances.

### Establishing ways of working

We recognise the importance of establishing good working relationships and clear ways of working with the CLAHRC communities. During the early stages of this project, we are working with CLAHRCs to agree on ways of working and have developed a memorandum of understanding to which each party is happy to commit (see Additional File 5).

### Development of evaluation framework and mapping mechanism-context-outcome links

In order to explore and describe the links between research and its implementation a 'theoretical map' of what CLAHRCs have planned concerning implementation is needed, which is incorporated into the study's evaluation framework. We will collect documentary evidence such as strategy documents, proposals and implementation plans, and other evidence. Drawing on the research implementation literature, we will discuss implementation and internal evaluation plans with each CLAHRC. Once gathered, we will analyse and synthesise the data using concept mining, developing analytical themes and framework development. The framework will yield what approaches and mechanisms each CLAHRC intends to be used for implementation, in what settings, with whom and to what affect.

Using the output of the documentary analysis, we will hold discussions with relevant stakeholders (*i.e.*, CLAHRC participants, NHS staff linked to CLAHRC projects, service user group, research team) to develop and refine MCO links, *i.e.*, the evaluation's hypotheses (for example, 'The translation and utilisation of knowledge in and through CLAHRCs and the resulting range of impacts will be dependent upon the different types of knowledge that are given attention and valued' and 'The impact of translation and implementation of knowledge in and through CLAHRCs will be dependent upon the adoption and use of appropriate facilitation approaches, including individuals in formal and informal roles'). We will then ensure that the hypotheses are shared across all nine CLAHRCs. This will provide another opportunity to scrutinise the credibility and representativeness of our hypotheses across contexts, and also to share knowledge that could be used more widely by CLAHRC programme participants.

### Tracer issues

To provide a focus for testing the hypotheses, we will work with the three CLAHRCs to determine what topics would be appropriate to become tracer issues. Criteria of choice will include the potential to have greatest impact in practice, examples from the increased uptake of existing evidence as well as new evidence being generated through CLAHRCs, and that might provide the most useful formative information for CLAHRCs and summative data for this evaluation. We anticipate that at least one of the tracer issues will be common to all three CLAHRCs to enable greater comparison.

Using available documents and our discussion with CLAHRC teams, we will map the clinical and implementation issues being addressed within and across each CLAHRC. Once these have been mapped, we will reach consensus with them about which topics become tracer issues. Tracer issues may not necessarily be clinical issues, but it is likely that the projects we focus on for in-depth study will have a particular clinical focus (*e.g.*, nutrition care, diabetes, stroke, kidney disease, long-term conditions). For example, one tracer issue could be change agency, the focus of in-depth study within a particular CLAHRC could then be the role of knowledge brokering in the implementation of improved service delivery for patients with chronic kidney disease.

### Phase two: Studying research implementation over time-testing hypotheses (up to 28 months)

We will test the hypotheses developed in phase one against what happens in reality within each CLAHRC case and tracer issue (*i.e.*, what is working (or not), for whom, how, and in what circumstances) over time. We will focus on specific projects/initiatives/activities within the tracer issues and conduct in-depth case studies on these.

To facilitate description, explanation, and evaluation, within each site multiple data collection methods will be used in order to identify different impacts or types of knowledge use as shown in Additional File 6. During phase one, we will negotiate the details and timings of phase two data collection activity, which will be dependent on the stages of CLAHRC development and other factors that are influencing CLAHRCs (*e.g.*, health service re-organisations). Being guided by our evaluation framework, objectives, and MCOs, we will aim to capture data at critical points in the implementation pathways of tracer issues. We plan for data collection and analysis to be iterative and cyclical; checking our observations against MCOs, and feeding this information back to participating sites as formative input (what seems to be working (or not), for whom, how, and in what circumstances). There will be four rounds of data collection and MCO refining over 28 months.

We will draw on the following data collection methods as appropriate for each in-depth study.

### Interviews

We will conduct semi-structured interviews with stakeholders at multiple levels within and across the particular project/initiative (*e.g.*, role of knowledge brokering in the implementation of improved service delivery for patients with chronic kidney disease). A sampling framework for interviews will be developed based on a stakeholder analysis [83]. Using both theoretical and criterion sampling, we will determine which stakeholders are 'essential,' 'important,' and/or 'necessary' to involve [78]. We will commence interviews with a representative sample of essential stakeholders, and further stakeholders will be interviewed from the other two categories based on theoretical sampling. Criterion sampling will be used to ensure the inclusion of a variety of stakeholders with criteria being developed to include different roles, length of involvement for example, in CLAHRCs.

Interviews will focus on perceptions about what is influencing implementation efforts, the content of which will be informed by MCOs and evaluation framework, as well as participant-driven issues. We are interested in exploring stakeholder perceptions of both the intended and unintended consequences or impact of implementation. As appropriate, interviews will be conducted either face-to-face or by telephone, and will be audio-recorded. The number of interviews conducted will be determined on a case-by-case basis, but is likely to be up to 20 in each case studied at each round of data collection.

### Observations

Focussed observation of a sample of tracer issue community of practice activities and team interactions (*e.g.*, between implementers and users, planning and implementation meetings) will be undertaken at appropriate points throughout this phase. We will identify a range of 'events' that could be observed and map these against our objectives to identify appropriate sampling. These observations will focus on interactions and be informed by an observation framework developed from Spradley's [84] nine dimensions of observation, including space, actors, activities, objects, acts, events, time, goals, and feelings. Observations will be written up as field notes.

### Routine and project-related data

As appropriate to the topic and outcomes of interest, we will draw on data being gathered by CLAHRCs, which they are willing to share. It is difficult to anticipate which data may be informative at this stage, but it could include implementation plans, ethics and governance applications, findings from specific implementation efforts and measures of context, minutes of meetings, internal audit data, cost data, and evidence of capacity and capability building (*e.g.*, research papers, staff employment, new roles, research activity). We will negotiate access to such information on a case-by-case basis.

### Publicly available data

Because CLAHRCs are regional entities and over time their impact might be realised at a population level, publically available information relevant to the tracer issues from Public Health Observatories and the Quality and Outcome Framework for general practitioners (for example) in participating CLAHRC areas could be a useful source of information. These data could be mined and tracked over time, and compared to data from non-CLAHRC areas; specifically, we are interested in exploring data from regions that were not successful in the CLAHRC application process. Whilst we recognise there will be a time lag in realising an impact of CLAHRC activity, these data have the potential to help our understanding about the effect of CLAHRCs on population health outcomes.

### Documents

We will gather and analyse documentary material relevant to: implementation, generally in relation to CLARHC strategy and approaches, and specifically with respect to the tracer issue and related project/initiative' context of implementation (*e.g.*, about wider initiatives, success stories, critical events/incidents, outputs, changes in organisation.); and CLAHRC internal evaluation plans. These materials may include policies, minutes of meetings, relevant local/national guidance, research/development/quality improvement papers, newspaper stories, job adverts, and reports (*e.g.*, about the CLAHRC programme more widely). These will provide information with which to further contextualise findings, provide insight into influences of implementation, and help explanation building.

### Evaluation team reflection and monitoring

Including key CLAHRC staff as research collaborators and the provision of formative learning opportunities will enable CLAHRCs to critically review (and potentially adapt) their implementation strategy and activities. In this respect, knowledge will be produced within a context of application, which requires nuanced approaches to establishing research quality [85]. The insider perspective from members of the research team will provide additional insights and enable us to crosscheck face validity of findings against the experience of operating within a CLAHRC context. A range of benchmarks (*e.g.*, immersion in the field, member-checking, audit trail) are available to demonstrate transparency in the interpretation of study findings. However, additional strategies to establish research quality are required that accommodate for the (potential) adaptation of CLAHRC's implementation programmes occurring through the cycle of learning and teaching described earlier. An information management strategy (including accurate record keeping, document version control, and information flow charts) will be established to allow a real time record of (codifiable) information sharing within the research team and with CLAHRCs. Once information flows are established, then it will be possible to explore the impacts of specific information sharing (*e.g.*, progress reports) in targeted interviews. Research team meetings will provide an important opportunity to adopt a reflexive approach to the discussion of the potential and actual impacts of findings within CLAHRCs through recording and observations of these meetings, and the maintenance of an evaluation team critical event diary. We will take a reflexive approach to meetings and ensure consideration of how our approach and/or contact may have influenced CLAHRC activity. As metadata, this information will be used in two ways: as a contribution to understanding implementation processes and influences; and to evaluate our decisions and actions to better understand how to conduct evaluations such as this in the future.

### Phase three: Testing wider applicability (up to six months)

Closing the realistic evaluation loop (Figure 2), we will test the wider applicability of findings emerging from phases one and two (see section below for analysis process) with a wider community. We will hold a joint interpretative forum-an opportunity for different communities to reflect on and interpret information from data collection efforts-enabling the surfacing of different viewpoints and knowledge structures for collective examination [86].

Members from relevant communities, including participants from all nine CLAHRCs, representatives from other initiatives such as Academic Health Science Centres, researchers and practitioners, service user representatives, policy makers, funders, commissioners, and managers interested in research implementation and impact will be invited. We will use our international networks to broaden the scope of attendance beyond the UK.

Using interactive methods and processes, and facilitated by an expert, we will test out our emerging theories about what works, for whom, how, and in what circumstances. Participants will be given the opportunity to challenge and interpret these from the position of their own frame of reference. We will capture workshop data through appropriate multimedia, such as audio recording, images, and documented evidence. These data will be used to refine theory.

This phase will provide an opportunity to maximize the theoretical generalisability of findings, will serve as a knowledge-transfer activity, and provide an opportunity to develop the potential for international comparison. The outputs of the forum will also be translated into a web-based resource for open access.

### Data analysis

The focus of analysis will be on developing and refining the links between mechanisms, context and outcomes (*i.e.*, hypotheses testing and refining) to meet study objectives. As a multi-method comparative case study, we will use an analysis approach that draws on Yin [81], Miles and Huberman [87], and Patton [82]. As this is a longitudinal evaluation, teasing out MCO configurations/interactions will involve an ongoing process of analysis, and be undertaken by various members of the team to ensure the trustworthiness of emerging themes. For each MCO, evidence threads will be developed from analysing and then integrating the various data; the fine-tuning of MCOs is a process that ranges from abstraction to specification, including the following iterations.

We will develop the theoretical propositions/hypotheses (with CLAHRCs in phase one around objectives, theories, and conceptual framework)-these MCOs are at the highest level of abstraction-what might work, in what contexts, how and with what outcomes, and are described in broad/general terms, *e.g.*, 'CLAHRC partnership approach' (M_1_), is effective (O_1_) at least in some instances (C_1_, C_2_, C_3_).

As data are gathered through phase two, data analysis and integration facilitates MCO specification ('testing') that will be carried out in collaboration with CLAHRCs. That is, we will refine our understanding of the interactions between M_1, _O_1, _C_1_, C_2_, and C_3_. For example, data analysis shows that in fact there appear to be particular approaches to partnerships (now represented by M_2_), that have a specific impact on increased awareness of research evidence by practitioners (now represented by O_2_), only in instances in teams where there is multi-disciplinary working (an additional C, now represented by C_4_). This new MCO configuration (*i.e.*, hypothesis) can then be tested in other settings/contexts/sites seeking disconfirming or contradictory evidence.

Cross-case comparisons will determine how the same mechanisms play out in different contexts and produce different outcomes. This will result in a set of theoretically generalisable features addressing our aims and objectives.

Consistent with comparative case study each case is regarded as a 'whole study' in which convergent and contradictory evidence is sought and then considered across multiple cases. A pattern matching logic, based on explanation building will be used [81,87]. This strategy will allow for an iterative process of analysis across sites, and will enable an explanation about research implementation to emerge over time, involving discussions with the whole team. Analysis will first be conducted within sites, and then to enable conclusions to be drawn for the study as a whole, findings will be summarised across the three sites [81,82]. Our evaluation and theoretical framework will facilitate data integration.

### Ethical issues

While some ambiguity exists in relation to the definitions of quality improvement, implementation research, and evaluation projects in relation to the need for formal ethical approval [88,89], this study will be generating primary data. Following the principles of good research practice [90,91], ethical approval will be sought from a multi-site research ethics committee for data collection from phase two onwards. The nature of the evaluation as an iterative and interactive process may necessitate a phased application to research ethics in order to provide the necessary detail for each round of data collection.

In line with good research practice [92], we will adhere to the following principles.

## Consent

Whilst CLAHRCs as a whole are contractually obliged to engage in external evaluation activities, the participation of individuals in this study is voluntary. Participants will be provided with written information about the evaluation and details of the nature and purpose of the particular data-collection activities before being asked to provide written consent to participate. They will have the right to withdraw consent at any point without giving a reason. We recognise that in research of this nature, there is always scope for exposing issues of concern, for example, poor quality of practice or service failings. Should issues of this nature occur in the course of data collection, the participant would be made aware that the researcher, following research governance and good research practice guidance [90-92], would discuss these in the first instance with the study principal investigator and further action taken as necessary.

### Confidentiality and anonymity

Participants will be known to the researchers gathering primary data, but beyond this, they will be assigned codes and unique identifiers to ensure and maintain anonymity. Where individuals are recognisable due to information provided in, for example, audio-recorded interviews, at the point of transcription a process of anonymising will be used to ensure that they are not recognisable. As it may be possible to identify staff who hold unique or unusual roles if their job title were used in the written reporting of data, alternative ways of recording these will be used, such a providing a general title to protect their anonymity. Details of the codes will be stored according to good practice and research governance requirements [90,91].

### Data management and storage

Documentary data, interview transcriptions, and fieldwork diaries will be stored securely. Only the principal investigator and research fellow will have access to primary data. Back-up copies of interviews will be stored separately, but in the same manner and all data kept on a password-protected computer.

### Burden

There have been discussions with CLAHRC directors at an early stage about ensuring burden and disruption are minimised, and this has been formalised in the memorandum of understanding (see additional file 5). We will therefore negotiate and agree the practicalities of data collection at each phase and round of data collection at a local level. Our study design allows us to take a flexible approach with the potential for amendment as necessary to reflect changing circumstances in each CLAHRC. Wherever possible, our evaluation will complement those being undertaken internally by each CLAHRC and with the three other NIHR SDO Programme evaluation teams.

## Discussion

The rationale underpinning the investment in the CLAHRC initiative and the theory on which they have been established is that collaboration between academics and practitioners should lead to the generation of more applied research, and a greater chance that research will be used in practice [13]. Despite a growing interest and belief in this theory [93], it has yet to be fully tested. This study has been designed to explore the unknown, as well as build on what is already known about research implementation within a collaborative framework through a theory and stakeholder driven evaluation.

Currently there are plans for a radical change in the way that healthcare is commissioned, planned, and delivered within the NHS [94]. Policy changes will mean fundamental shifts to the way some CLAHRCs are managed and funded, which have the potential to create a very different context for them, and a significantly different evaluation context for us. For example, the introduction of competition within a local health economy may result in fragmentation and a tendency to be less open and collaborative-the antitheses of the philosophy upon which CLAHRCs were established. Realistic evaluation provides an ideal approach for monitoring how such policy changes impact on CLAHRC over time. As the evaluation progresses and the MCOs are tested and refined, we will pay attention to the impact that these wider political changes have in terms of acting as barriers or enablers to knowledge generation, implementation, and use.

In addition, the local response to the current governmental debate about NHS funding as one aspect of widespread public sector revisions, is as yet unknown. It is inevitable that in a time of financial austerity the CLAHRCs will face challenges about how they interpret and manage decisions about their joint remit for research and implementation. This, in turn, may impact on our evaluation, depending on the nature and extent of, for example, reductions, amendments, or cessation of the planned projects undertaken in the CLAHRCs. A pragmatic and flexible approach to undertaking research in 'real world' settings, and in particular in health care, is increasingly recognised as not only realistic, but necessary [95].

As described earlier, this is a longitudinal and interactive evaluation, which has some potential advantages. Realistic evaluation is iterative and engages stakeholders throughout the process. This will ensure we are able to adapt to ongoing changes to circumstances and facilitate the development of robust and sustained working relationships with the CLAHRCs. Engaging CLAHRC members in the development of the proposal and ongoing delivery of the research should ensure an appropriately focussed evaluation, contextually sensitive approaches to data collection, and opportunities for sharing and verifying emerging findings.

This evaluation was funded to provide information for learning, not for judgement. The purpose of the evaluation is formative, focusing on processes and a range of potential and actual impacts from implementation and use of knowledge as they occur over the lifespan of the evaluation and beyond the initial funding period of the CLAHRCs (2008 to 2013). The outputs of the study will be both theoretical and practical, and therefore opportunities for formative learning have been built in.

There are a number of ways the findings from this evaluation may contribute to knowledge about implementation. CLAHRCs provide a rare opportunity to study a natural experiment in real time, over time. The idea that collaboration, partnership, and sustained interactivity between the producers and users of knowledge lead to the production of more applicable research and increases the likelihood that research will be used in practice, has grown in popularity within the implementation science healthcare community. Whilst this is the theory, in practice we do not know whether this is the case, what the facilitators and barriers are to this way of working, or what the intended and unintended consequences may be. Our evaluation is designed to capture the processes and impacts of collaborative approaches for implementing research in practice, and therefore should contribute to the evidence base about an increasingly popular (*e.g.*, mode two, integrated knowledge transfer, interactive research), but poorly understood approach to knowledge translation. Additionally, we have specific research questions about the role particular collaborative mechanisms, such as communities of practice and boundary objects play. Addressing these questions has the potential to increase our understanding of these mechanisms as potential implementation interventions, and inform future evaluation studies.

To date, much of the research exploring implementation processes and impacts has been conducted with a focus on isolated and one-off projects or initiatives, such as the implementation of a guideline or procedure. This means that we know little about implementation within sustained and organisational initiatives. As a longitudinal study that is focused at multiple levels within large regional entities, this evaluation could add to what we know about organisation level implementation initiatives over a sustained period of time.

Finally, we hope to contribute to methods for evaluating implementation processes and impacts. We have described why realistic evaluation is appropriate for this study; however, there are limited examples of its use in the published literature. This is an ideal opportunity to apply, and potentially develop, this approach, particularly with respect to integrated stakeholder involvement.

### Study limitations

Case study research generates findings that are theoretically transferrable to other similar settings, but does not provide generalisable data, and therefore trying to generalise findings to other contexts either in the UK or in international settings should be undertaken with caution and acknowledgement of its provenance.

Each data collection method has its own limitations, but the benefit of using several data sources as triangulation of methods can largely overcome these by providing multiple perspectives on phenomena. To enhance the trustworthiness of data, the researchers will use a reflective approach to conducting the study, and this will be further explored and recorded as part of the project learning.

## Competing interests

The authors declare that they have no competing interests.

## Authors' contributions

JR-M is the principal investigator for the study. She conceived, designed, and secured funding for the study in collaboration with CB, RB, SD, GH, IG, SS, CT, BM, and GA. JRM wrote the first draft of the manuscript with support and input from JW and CB. All authors (SA, GA, CB, RB, SD, GH, IG, SS, CT, GM, BM, and JW) have read drafted components of the manuscript, provided input into initial and final refinements of the full manuscript. All authors read and approved the final submitted manuscript.

## Supplementary Material

Additional file 1 **CLAHRCs - the concept**. Background to CLAHRCs Click here for file

Additional file 2 **South Yorkshire CLAHRC**. Background to South Yorkshire CLAHRC Click here for file

Additional file 3 **Greater Manchester CLAHRC**. Background to Greater Manchester CLAHRC Click here for file

Additional file 4 **Leicester, Northamptonshire and Rutland CLAHRC**. Background to Leicester, Northamptonshire and Rutland CLAHRC Click here for file

Additional file 5 **MOU**. Memorandum of Understanding Click here for file

Additional file 6 **Summary of Data Collection Activity**. Includes Objectives, Phase, Methods, Type of impact and outcomes Click here for file
