# Supplementary material for: Implementing health research through academic and clinical partnerships: a realistic evaluation of the Collaborations for Leadership in Applied Health Research and Care (CLAHRC)
Source: Implement Sci. 2011 Jul 19;6:74. doi: 10.1186/1748-5908-6-74 (PMC3168414; doi:10.1186/1748-5908-6-74)
Supplement: Additional file 1 — CLAHRCs - the concept. Background to CLAHRCs [file 1748-5908-6-74-S1.DOC]

Additional file 1
Title: CLAHRCs – the concept
Description: Background to CLAHRCs

**Box 1. CLAHRCs – the concept**

**Background to CLAHRCs**

To provide a specific response to the key recommendations of Chief Medical Officer’s high level Clinical Effectiveness group [96]) which recognised that many key advances in medical practice have resulted from partnerships between academia and the NHS and that some approaches such as educational outreach and academic detailing have shown promise in the delivery of clinical effectiveness. In addition, there is scope for new ways of working together to address the shift to greater care provision in the community and the focus on public health and the management of long term conditions.

There is evidence that a major predictor of the application of research to practice is the extent that researchers producing research and practitioners who will use the findings in practice interact throughout the research process [8]. This is in essence the *raison d’être* of the CLAHRCs, to provide an environment where researchers and practitioners can work together in this way.

CLAHRCs provide the opportunity to harness the capacity of higher education institutions to assist with this agenda by promoting new models of community-wide ‘academic health centres’ to encourage relevant research, engagement, and a population focus, and embed a critical culture that is more receptive to change. The development of new interventions would include an analysis of the mechanisms of implementation [13].

**Purpose of CLAHRCs**

‘To forge a mutually beneficial, forward looking partnership between a university and the surrounding NHS organisations focused on improving patient outcomes through the conduct and application of applied health research’ [13]. Applied health research is seen as research that has a practical application for the benefit of patients, through improved health care or delivery, where there is potential within a relatively short time scale (three to five years) [13].

**Aims of CLAHRCs**

The aims of CLAHRCs are:

1. To develop innovative models for conducting applied health research and translating research findings into improved patient outcomes.
2. To create a distributed model across a health community (geographical area of a CLAHRC) for the conduct and application of research for all those who will use it in practice.
3. To create and embed approaches to research and dissemination that are designed specifically to take account of the way health care is delivered across sectors and geographical areas.
4. To increase the country’s capacity for conducting and implementing high-quality applied health research that focuses on the needs of patients, in particular those with chronic disease and relating to public health interventions bounded by the geographical area of the CLAHRC.
