# Supplementary material for: Implementing health research through academic and clinical partnerships: a realistic evaluation of the Collaborations for Leadership in Applied Health Research and Care (CLAHRC)
Source: Implement Sci. 2011 Jul 19;6:74. doi: 10.1186/1748-5908-6-74 (PMC3168414; doi:10.1186/1748-5908-6-74)
Supplement: Additional file 2 — South Yorkshire CLAHRC. Background to South Yorkshire CLAHRC [file 1748-5908-6-74-S2.DOC]

Additional file 2
Title: South Yorkshire CLAHRC
Description: Background to South Yorkshire CLAHRC

**South Yorkshire CLAHRC**

Lead Organisation: Sheffield Teaching Hospitals NHS Foundation Trust.

Academic Partner: University of Sheffield and Sheffield Hallam University

Other organisations involved: Sheffield Hospitals Charitable Trust and related charities; NHS Yorkshire and Humber; NHS Doncaster and Bassetlaw Hospitals Foundation Trust; NHS Sheffield; NHS Rotherham Foundation Trust; NHS Doncaster; NHS Barnsley; NHS Sheffield Children’s Foundation Trust; Sheffield Health and Social Care Foundation Trust; South Yorkshire NIHR Comprehensive local research network, Medipex Ltd; NHS Yorkshire and the Humber; Research Design Service Yorkshire and the Humber; Rotherham, Doncaster and South Humber Mental Health Foundation Trust.

**Vision and mission statements**

‘Our vision is for South Yorkshire to become internationally recognised in the field of self-management of long term conditions through applied research, health technology innovations and the translation of knowledge into quality patient care.’

‘Our mission is to undertake, over the next five years, high quality, strategic, applied research and related education in order to enable a ‘step change’ in the way research is delivered and services are designed in South Yorkshire; and to foster knowledge transfer that will improve the quality and effectiveness of health care delivery across South Yorkshire.’ (Source : SY CL brochure, undated)

SY CL has separated research and implementation activity but both address a number of clinical and other themes. Evaluation of these will be undertaken by an internal evaluation team (Source : www.clahrc-sy.nihr.ac.uk: Accessed 08/04/10).

| **Theme** | **No. of research projects** | **No. of implementation projects** |
| --- | --- | --- |
| COPD | 0 | 4 |
| Core activities including internal evaluation | 0 | 3 |
| Diabetes | 2 | 1 |
| Depression | 2 | 0 |
| Genetics | 2 | 2 |
| Knowledge to Action | 0 | 3 |
| Health inequalities | 0 | 2 |
| Intelligent commissioning | 0 | 1 |
| Obesity | 2 | 0 |
| Telehealth and care technologies | 1 | 2 |
| Stroke | 6 | 3 |
| User centred health care design | 0 | 1 |

Examples of ongoing projects: (Source: clahrc-sy.nihr.ac.uk: Accessed 08/04/10)

Obesity (Research): CLAHRC funding will be used to set up a cohort to be tracked over the lifespan of the CLAHRC with the primary purpose of informing NHS provision of healthcare with regards to self-management, and management and the effect of particular variables on health outcomes.

COPD (Implementation): Rotherham Breathing Space Programme: This is a community-based multi-disciplinary COPD rehabilitation programme providing in-patients and day-patients with pulmonary rehabilitation and respite care, supporting patients in self-management of their condition. The CLAHRC is supporting the extension of this programme (2009-2013) to facilitate research into the long term whole system impact of the programme.
