# Supplementary material for: Implementing health research through academic and clinical partnerships: a realistic evaluation of the Collaborations for Leadership in Applied Health Research and Care (CLAHRC)
Source: Implement Sci. 2011 Jul 19;6:74. doi: 10.1186/1748-5908-6-74 (PMC3168414; doi:10.1186/1748-5908-6-74)
Supplement: Additional file 3 — Greater Manchester CLAHRC. Background to Greater Manchester CLAHRC [file 1748-5908-6-74-S3.DOC]

Additional file 3
Title: Greater Manchester CLAHRC
Description: Background to Greater Manchester CLAHRC

**Greater Manchester CLAHRC (GM CL)**

Lead NHS Organisation: NHS Salford with Salford Royal Foundation Trust hosting the NHS staff working on the CLAHRC.

Academic organisation: University of Manchester

Partner organisations:

NHS Ashton Leigh & Wigan; NHS Bolton; NHS Bury; NHS Heywood Middleton & Rochdale; NHS Manchester; NHS Oldham; NHS Salford; NHS Stockport; NHS Tameside & Glossop; NHS Trafford; Greater Manchester West NHS Mental Health Foundation Trust; Manchester Mental Health & Social Care Trust; Pennine Care Mental Health Trust; Bolton Hospitals NHS Trust; Central Manchester University Hospital NHS Foundation Trust; Pennine Acute Hospital NHS Trust; Stockport NHS Foundation Trust; University Hospitals of South Manchester NHS Foundation Trust; North West Ambulance Service Trust.

Objectives of GM CL: ‘The CLAHRC’s five year mission is to improve health care and reduce inequalities in health for people with long term vascular conditions by developing and evaluating improved ways for the NHS to support people in managing their vascular conditions. Implementing these and other evidence-based improvements in care in the NHS across Greater Manchester and by building capacity to plan and implement evidence-based changes to care pathways for people with vascular conditions.’ (Source GM CL brochure, undated)

GM CL has separated research and implementation activity but both address a number of clinical and other themes: (Source GM CL brochure, undated). Evaluation is integrated into each of these, rather than being undertaken as a separate activity on GM CL.

| **Theme** | **Research** | **Implementation** |
| --- | --- | --- |
| People with long term conditions | √ |  |
| Health care practitioners | √ |  |
| Health care services | √ |  |
| Health information systems | √ |  |
| Stroke |  | √ |
| Diabetes |  | √ |
| Chronic kidney disease |  | √ |
| Heart disease |  | √ |

Examples of ongoing projects: (Source: clahrc-gm.nihr.ac.uk: Accessed 08/04/10

Health Care practitioners theme (Research) is developing better approaches to the management of depression in patients with vascular conditions.

Chronic Kidney Disease (CKD) theme (Implementation) is using the Institute for Healthcare Improvement’s Breakthrough Series Collaborative methodology to work with general practice teams to improve the identification and management of CKD, supported by improvements in staff education, leadership, information and patient involvement.
