# Supplementary material for: Implementing health research through academic and clinical partnerships: a realistic evaluation of the Collaborations for Leadership in Applied Health Research and Care (CLAHRC)
Source: Implement Sci. 2011 Jul 19;6:74. doi: 10.1186/1748-5908-6-74 (PMC3168414; doi:10.1186/1748-5908-6-74)
Supplement: Additional file 4 — Leicester, Northamptonshire and Rutland CLAHRC. Background to Leicester, Northamptonshire and Rutland CLAHRC [file 1748-5908-6-74-S4.DOC]

Additional file 4
Title: Leicester, Northamptonshire and Rutland CLAHRC
Description: Background to Leicester, Northamptonshire and Rutland CLAHRC

**Leicester, Northamptonshire and Rutland CLAHRC (LNR CL):**

Lead Organisation: University Hospitals of Leicester NHS Trust

Academic Partner: University of Leicester

Partner organisations:

Northamptonshire General Hospitals NHS Trust; Kettering General Hospitals NHS Foundation Trust; Leicester Partnership Trusts; Northamptonshire Health Care NHS Foundation Trust; NHS Leicester City; NHS Leicester County & Rutland; NHS Northamptonshire; University of Leicester East Midland Workforce Deanery; NHS East Midlands.

Mission statement: ‘Working together to conduct high quality research to improve the health of the population of Leicester, Northamptonshire and Rutland.’ ‘A five year programme to ensure lessons are learned from research studies are rapidly and effectively implemented and to develop research capacity and capability with local healthcare organisations.’ (Source: clahrc-lnr.nihr.ac.uk: Accessed 08/04/10).

LNR CL has separated research and implementation themes (Source: clahrc-lnr.nihr.ac.uk Accessed 10/04/10). Evaluation of these will be undertaken by an internal evaluation team.

| **Theme** | **Research** | **Implementation and Translation** |
| --- | --- | --- |
| Early Detection | Five studies |  |
| Prevention | Three studies |  |
| Self Management and Education | Number not detailed |  |
| Rehabilitation | Three studies |  |
|  |  | Eight studies detailed with more planned |

Examples of ongoing projects (Source: clahrc-lnr.nihr.ac.uk Accessed 10/04/10 & 05/01/11).

LNR CL view implementation and translation as two separate processes [12].

PSP-CKD Study (Research): A study looking at preventing barriers to treating patients with and at risk of chronic kidney disease

Implementation of NICE Guidelines on Obesity (Implementation): It is hoped that this project will assist in understanding how implementation can be made more consistently effective. Practices and PCTs will also benefit, receiving a package to help in implementing the NICE guideline on obesity.

Explaining Acute Admissions (Translation): to explain why the observed acute admission rate in NHS Leicester County & Rutland is higher than expected from appropriately adjusted national data. This multi-method project will provide the Trusts with information about the reasons for high acute admission rates. This will lead on to proposals for means of reducing admission rates.
