# Supplementary material for: Implementing health research through academic and clinical partnerships: a realistic evaluation of the Collaborations for Leadership in Applied Health Research and Care (CLAHRC)
Source: Implement Sci. 2011 Jul 19;6:74. doi: 10.1186/1748-5908-6-74 (PMC3168414; doi:10.1186/1748-5908-6-74)
Supplement: Additional file 5 — MOU. Memorandum of Understanding [file 1748-5908-6-74-S5.DOC]

Additional File 5

**Memorandum of Understanding**

**SDO EVALUATION OF CLAHRCS**

***‘CLAHRCS in Action, Theory, Process and Impact.’***

**Memorandum of Understanding**

**September 2010**

**Prof Jo Rycroft-Malone, Bangor University, Wales.**

**Dr Steven Ariss (SY CLAHRC)**

**Dr Christopher Burton**

**Prof Sue Dopson**

**Prof Ian Graham**

**Dr Gill Harvey (GM CLAHRC)**

**Dr Graham Martin (LNR CLAHRC)**

**Prof Brendan McCormack**

**Dr Sophie Staniszewska**

**Prof Carl Thompson**

**Dr Joyce Wilkinson (Research Fellow)**

**Dr Gavin Andrews (Collaborator)**

*Purpose:*

This memorandum of understanding (MoU) has been developed to outline ways of working between the SDO Evaluation Team (P.I Prof Jo Rycroft-Malone) and the CLAHRC teams, specifically, but not exclusively, the three CLAHRCs that are the main focus of this evaluation, South Yorkshire (SY); Greater Manchester (GM) and Leicester, Northamptonshire and Rutland (LNR). The evaluation commenced on 6^TH^ January 2010 and will end on 31^st^ March 2014.

*Contents:*

It outlines broad principles and ways of working and details how this will be achieved. It also includes details of action to be taken in the event of any problems or difficulties that arise in the course of the research.

1. **Our responsibilities as a research team** undertaking this evaluation are:

- To undertake this evaluation as outlined in the research protocol accepted by NIHR/SDO as funders of the project. Grant No: 09/1809/1072.
- To undertake the evaluation according to the guidance provided by research governance and to the research protocol that will be submitted in due course and approved by Research Ethics Committees and local Research and Development departments within the CLAHRCs. We will also take account of other, wider principles such as those contained in the Good Clinical Practice guidance provided by NISCHR/NIHR and those of our professional Codes of Conduct.

1. **To work in an open and collaborative manner** with the CLAHRCs with the project team members representing each of the CALHRCs, as a first point of contact. These are:

- Dr Steven Ariss - SY CLAHRC
- Dr Gill Harvey – GM CLAHRC
- Dr Graham Martin – LNR CLAHRC

Contact with the CLAHRC Directors, their Deputies and other leads will be ongoing throughout the evaluation through formal and informal meetings and contacts; as and when necessary and appropriate. This two-way process will be augmented through the formal reporting arrangements to each of the CLAHRCs and to NIHR/SDO, and additional updates in the regular meetings with the CLAHRCs and other evaluation teams.

1. **To work in a participatory and co-operative manner** with the CLAHRCs and service users being mindful of the burden of work, and through co-operation with the other externally funded evaluation teams to minimise disruption and duplication for the CLAHRCs staff, whenever possible. We will achieve this by ongoing dialogue with CLAHRCs through the project team and Directors or their Deputies or nominated others, as appropriate. Clear written, email and verbal communication are fundamental to maintaining this two-way process.
2. **Responsibilities of the CLAHRCs in the evaluation**:

- To work with the evaluation team in a reciprocal collaborative and co-operative manner
- To support the team to have reasonable access to data and facilitate/mediate access for data gathering purposes, and when this is not possible, to discuss the reasons for this at the earliest opportunity with the P.I. and to work to reach a mutually acceptable solution.

1. **Intellectual Property and publications**: any potential publications will be discussed at an early stage of planning, as outlined in the Guidelines for Authorship (SDO Evaluation Team Author Guidelines July 2010) agreed with the project team. It will be the responsibility of the project team member from each of the CLAHRCs to discuss the guidelines and publications more widely with the appropriate staff within their CLAHRC and to feedback any issues or concerns at the earliest opportunity.

Our approach will seek to be inclusive and open in the process of agreeing joint publications and in the use of data or other information from the CLAHRCs gained through the evaluation, for, for example, presentations. In addition:

- Concerns about confidentiality should be raised by either the CLARHC or the evaluation team at the earliest opportunity and a way forward discussed, agreed and recorded.
- If there are issues relating to the need for anonymity of information that would/could identify a CLAHRC in relation to any presentations, reports or publications, this should likewise be raised at the earliest opportunity, and progressed in the same manner as the above point.
- Where there is the potential for any commercially sensitive information arising in the CLAHRCs and noted in the course of the evaluation, this will be discussed, again at the earliest opportunity between the CALHRC Director (or nominated deputy) and the Evaluation team P.I. Advice from Bangor University’s I.P manager will be sought if necessary to protect the interests of both the CLAHRC and the Evaluation team. In relation to the above three points, the Evaluation team will not share any information relating to a CLAHRC without previously having discussed this with the team representative and the CLAHRC Director or Deputy, if the project team representative considers this to be necessary. A record of the discussion and decision will be kept by both parties.

1. Conflict resolution: in the event of any disagreements or difficulties relating to any aspect of the conduct of the evaluation, the matter should be raised in the first instance with Prof Jo Rycroft-Malone as the project P.I. If it is not able to be resolved, it would then be discussed with Jill Fairbank, CLAHRC programme support manager and if it still remains unresolved, it would then be raised with a representative of the NIHR SDO Programme.
2. This MoU will be reviewed on an annual basis, or sooner if required and updated as necessary.
3. Agreement with the principles contained within this MoU:

To indicate that this MoU has been read and agreed with, please sign and date below. A copy containing all the signatures will be retained by the Evaluation team and should also be held by the Director of each CLAHRC.

| **Prof Sue Mawson** | **Director: SY CLAHRC** | **Date** |
| --- | --- | --- |
|  |  |  |
| **Dr Steven Ariss** | **Evaluation Team: SY CLAHRC** | **Date** |
|  |  |  |
| **Prof Richard Baker** | **Director: LNR CLAHRC** | **Date** |
|  |  |  |
| **Dr Graham Martin** | **Evaluation Team: LNR CLAHRC** | **Date** |
|  |  |  |
| **Prof Bonnie Sibbald** | **Director: GM CLAHRC** | **Date** |
|  |  |  |
| **Dr Gill Harvey** | **Evaluation Team: GM CLAHRC** | **Date** |
|  |  |  |
| **Dr Christopher Burton** | **Evaluation Team** | **Date** |
|  |  |  |
| **Prof Sue Dopson** | **Evaluation Team** | **Date** |
|  |  |  |
| **Prof Ian Graham** | **Evaluation Team** | **Date** |
|  |  |  |
| **Prof Brendan McCormack** | **Evaluation Team** | **Date** |
|  |  |  |
| **Dr Sophie Staniszewska** | **Evaluation Team** | **Date** |
|  |  |  |
| **Prof Carl Thompson** | **Evaluation Team** | **Date** |
|  |  |  |
| **Dr Joyce Wilkinson** | **Evaluation Team** | **Date** |
|  |  |  |
| **Dr Gavin Andrews** | **Evaluation Team (Collaborator)** | **Date** |
|  |  |  |

This MoU will be reviewed in September 2011.

END
