# Supplementary material for: Implementing health research through academic and clinical partnerships: a realistic evaluation of the Collaborations for Leadership in Applied Health Research and Care (CLAHRC)
Source: Implement Sci. 2011 Jul 19;6:74. doi: 10.1186/1748-5908-6-74 (PMC3168414; doi:10.1186/1748-5908-6-74)
Supplement: Additional file 6 — Summary of Data Collection Activity. Includes Objectives, Phase, Methods, Type of impact and outcomes [file 1748-5908-6-74-S6.DOC]

Additional file 6
Title: Summary of Data Collection Activity
Description: Includes Objectives, Phase, Methods, Type of impact and outcomes

**Summary of data collection activity**

| **Objectives*** | **Phase** | **Methods** | **Type of impact and outcomes** |
| --- | --- | --- | --- |
| To identify and track the implementation mechanisms and processes used by CLAHRCs and evaluate intended and unintended consequences (*i.e.*, impact) over time | 1,2,3 | Document analysis, telephone interviews, consensus development. Hypothesis generation. Interviews, observation, routine and publically available data analysis. Interpretive forum. | Direct (instrumental)  Conceptual  Symbolic  Process |
| To determine what influences whether and how research is used (or not) through CLAHRCs, paying particular attention to contextual factors | 2,3 | Interviews, observation, routine and publically available data, documents, evaluation team reflection, interpretive forum | Process  Conceptual  Symbolic |
| To investigate the role played by boundary objects in the success or failure of research implementation through CLAHRCs | 1,2,3 | Hypothesis generation, interviews, observation, document analysis | Process  Conceptual  Symbolic |
| To determine whether and how CLAHRCs develop and sustain interactions and communities of practice | 1,2,3 | Hypothesis generation, interviews, observation, document analysis | Process  Conceptual  Symbolic |
| To identify indicators that could be used for further evaluations of the sustainability of CLAHRC-like approaches | 3 | Hypothesis generation, interpretive forum- making sense of data collected in phases one and two, evaluation team reflection | Direct (instrumental)  Conceptual  Symbolic  Process |

*****Reflecting the dynamic nature of research implementation, our objectives are not mutually exclusive; data collection activity will inform, to a greater or lesser extent, each objective
